# Enhanced Proliferation of Porcine Bone Marrow Mesenchymal Stem Cells Induced by Extracellular Calcium is Associated with the Activation of the Calcium-Sensing Receptor and ERK Signaling Pathway

**DOI:** 10.1155/2016/6570671

**Published:** 2016-03-30

**Authors:** Jingjing Ye, Wei Ai, Fenglin Zhang, Xiaotong Zhu, Gang Shu, Lina Wang, Ping Gao, Qianyun Xi, YongLiang Zhang, Qingyan Jiang, Songbo Wang

**Affiliations:** ^1^College of Animal Science and National Engineering Research Center for Breeding Swine Industry, South China Agricultural University, Guangzhou 510642, China; ^2^Guangdong Provincial Key Lab of Agro-Animal Genomics and Molecular Breeding and ALLTECH-SCAU Animal Nutrition Control Research Alliance, South China Agricultural University, Guangzhou 510642, China

## Abstract

Porcine bone marrow mesenchymal stem cells (pBMSCs) have the potential for application in regenerative medicine. This study aims to investigate the effects of extracellular calcium ([Ca^2+^]_o_) on pBMSCs proliferation and to explore the possible underlying mechanisms. The results demonstrated that 4 mM [Ca^2+^]_o_ significantly promoted pBMSCs proliferation by reducing the G0/G1 phase cell percentage and by increasing the S phase cell proportion and the proliferation index of pBMSCs. Accordingly, [Ca^2+^]_o_ stimulated the expression levels of proliferative genes such as cyclin A2, cyclin D1/3, cyclin E2, and PCNA and inhibited the expression of p21. In addition, [Ca^2+^]_o_ resulted in a significant elevation of intracellular calcium and an increased ratio of p-ERK/ERK. However, inhibition of calcium-sensing receptor (CaSR) by its antagonist NPS2143 abolished the aforementioned effects of [Ca^2+^]_o_. Moreover, [Ca^2+^]_o_-induced promotion of pBMSCs proliferation, the changes of proliferative genes expression levels, and the activation of ERK1/2 signaling pathway were effectively blocked by U0126, a selective ERK kinase inhibitor. In conclusion, our findings provided evidence that the enhanced pBMSCs proliferation in response to [Ca^2+^]_o_ was associated with the activation of CaSR and ERK1/2 signaling pathway, which may be useful for the application of pBMSCs in future clinical studies aimed at tissue regeneration and repair.

## 1. Introduction

Bone marrow mesenchymal stem cells (BMSCs) not only regulate hematopoietic and other stem cells niches but also have multipotential capacities to differentiate toward osteocyte, chondrocyte, adipocyte, and myocyte [1], performing an important role in regenerative medicine, wound healing, and disease therapy [2]. Pigs exhibit similar structure and function to those of humans and have been widely used as a valuable model in biomedical research such as tissue engineering and cell therapy [3]. In addition to the studies on the isolation and differentiation capacities of porcine BMSCs (pBMSCs) [4, 5], the investigation on pBMSCs proliferation, which ensures that a large amount of pBMSCs is obtained, is very important. Thus, controlling the proliferation of pBMSCs is an attractive approach to determine the size of the pBMSCs pool and subsequently its possible influence on the maintenance of stem cell niches and multilineage differentiation potential.

Calcium ion, one of the most widely occurring second messengers, is an important cellular signaling component, which has been shown to play a pivotal role in controlling cell proliferation [6, 7]. Extracellular calcium ([Ca^2+^]_o_) modulates cell proliferation in various cells, such as myeloma cells [8], rat bone marrow-derived progenitor cells [9], osteoblasts [10], preadipocytes [11], and synovium-derived mesenchymal stromal cells [12]. [Ca^2+^]_o_ exerts its role in regulating cell proliferation either via calcium influx through calcium channels or by activating the calcium-sensitive receptor (CaSR) [13]. CaSR is a G protein-coupled receptor on plasma membrane which senses [Ca^2+^]_o_ [14]. Several studies have shown that CaSR is expressed in mesangial cells [15], osteoblasts [10], and preadipocytes [11] and plays a vital role in the regulation of cell proliferation [16].

[Ca^2+^]_o_ always leads to the increase of intracellular calcium ([Ca^2+^]_i_) and subsequent activation of the intracellular signaling pathway, which regulates cell proliferation. The MAPK signaling pathway, which consists of extracellular signal-regulated kinase 1/2 (ERK1/2), Jun kinase (JNK), and p38 MAPK, plays an important role in controlling cell proliferation in mammalian cells [17]. In particular, ERK1/2 is involved in the proliferation of various cells, such as kidney epithelial cells [18], smooth muscle cell [19], melanoma cell [20], and preadipocytes [11]. ERK elicits its role in cell proliferation possibly through its effect on the cell cycle transition from the G0/G1 phase to the S phase [21] and/or on the expression of cyclin D [22, 23].

Although many studies described that [Ca^2+^]_o_ is involved in the regulation of cell proliferation, the role of [Ca^2+^]_o_ in pBMSCs proliferation and the possible mechanisms underlying this process remain unclear. Thus, the present study was designed to investigate the effects of [Ca^2+^]_o_ on pBMSCs proliferation by determining the cell numbers, cell cycle progression, and expression levels of proliferative marker genes. In addition, we sought to explore the underlying mechanisms involved in this process, including the contribution of CaSR and the relevant intracellular signaling pathway. Our results revealed that the enhanced pBMSCs proliferation in response to [Ca^2+^]_o_ was associated with the activation of the CaSR and ERK1/2 signaling pathway.

## 2. Materials and Methods

### 2.1. Chemicals and Antibodies

Calcium acetate, CaSR antagonist NPS2143, calcium indicator Fluo 3-AM, and ERK kinase inhibitor U0126 were purchased from Sigma-Aldrich. Cell Counting Kit-8 (CCK-8) was purchased from Vazyme Biotech Co., Ltd. (Nanjing, China). DMEM/F12 and fetal bovine serum (FBS) were purchased from Gibco BRL. Polyclonal antibodies against *β*-actin, cyclin D1, ERK1/2, phospho-ERK1/2, and p21 were purchased from Cell Signaling Technology Inc. Polyclonal antibodies against CaSR were purchased from Abcam plc.

### 2.2. Cell Culture and Treatment

pBMSCs were isolated and purified from the bone marrow of postnatal Landrace pigs aged between 5 and 7 days as we previously described [4]. The purified pBMSCs were seeded in a 96-well plate with density of 5000 cells/well and cultured in DMEM/F12 medium (containing 1 mM calcium) supplemented with 10% FBS, 100 U/mL of penicillin sodium, and 100 *μ*g/L of streptomycin sulfate in a humidified cell incubator with atmosphere of 5% CO_2_ at 37°C. The pBMSCs were treated with various concentrations (1, 2, 4, and 6 mM) of [Ca^2+^]_o_ for 5 days to investigate the effects of [Ca^2+^]_o_ on pBMSCs proliferation. In addition, the cells were treated with 4 mM [Ca^2+^]_o_ and/or 0.1 *μ*M NPS2143 or 1 *μ*M U0126 for 5 days to explore the role of CaSR and ERK in [Ca^2+^]_o_-induced proliferation of pBMSCs, respectively.

### 2.3. Cell Proliferation Assay

After pBMSCs were cultured with different treatments for 5 days, the cells were then incubated with Cell Counting Kit-8 (CCK-8) reagent (0.1 mg/well) for 4 h. The number of viable cells was assessed by measuring the absorbance at 450 nm using a Synergy 2 Multi-Mode Reader (BioTek, USA).

### 2.4. Cell Cycle Analysis

pBMSCs were seeded at 1 × 10^6^ cells/per 25 cm^2^ flask and cultured in presence of 4 mM [Ca^2+^]_o_ and/or CaSR antagonist NPS2143 supplementation for 5 days. Cell cycle status was determined by measuring cellular DNA content following staining with propidium iodide using flow cytometry as previously described [24]. Briefly, the cells were centrifuged and washed twice with ice-cold phosphate-buffered saline (PBS) and then fixed overnight in 70% ethanol at 4°C. Fixed cells were centrifuged at 2500 rpm for 5 min and the supernatant was discarded. Pellets were washed twice and incubated with propidium iodide/RNase Staining Buffer (BD Pharmingen, USA) for 30 min at room temperature in the dark. DNA content was determined by flow cytometer (Gallios, Beckman Coulter, USA) with excitation at 488 nm and emission at 630 nm. The data were analyzed using MultiCycle for Windows software. Proliferation index (PI) was calculated according to the following equation: PI = (S + G2/M)/(G0/G1 + S + G2/M) × 100% [25].

### 2.5. Measurements of Intracellular Calcium

The pBMSCs, plated on a 12-well plate, were cultured with 4 mM [Ca^2+^]_o_ and/or CaSR antagonist NPS2143 for 12 h and then loaded with Fluo 3-AM (2 *μ*M) in the HEPES buffered salt solution (HBSS) (NaCl 137 mM, KCl 2.7 mM, NaH_2_PO_4_ 0.4 mM, CaCl_2_ 0.9 mM, 0.5 mM MgCl_2_, 10 mM HEPES, and 5.5 mM glucose, pH 7.4) for 30 min at 37°C. The loaded cells were rinsed three times with HBSS. Intracellular calcium ([Ca^2+^]_i_) levels were then determined by flow cytometry using a BD FACSAria2 flow cytometer (BD Bioscience, USA) at an excitation wavelength of 488 nm and an emission wavelength of 525 nm. Data analysis was performed using the FlowJo software.

### 2.6. Western Blot Analysis

At the end of incubation, the pBMSCs were harvested and washed twice with PBS. Then Western blot was conducted as previously described [26]. Cells were lysed by lysis buffer, and the cell lysates were centrifuged to remove insoluble materials and the protein concentration of each sample was measured. Equal protein amounts of each sample were separated by SDS-PAGE and electroblotted to PVDF membranes (Millipore, Billerica, MA, USA). The membranes were blocked with 5% nonfat milk in TBST for 2 h at room temperature and then incubated with different primary antibodies, including anti-CaSR (1 : 500), anti-cyclin D1 (1 : 2000), anti-p21 (1 : 2000), anti-ERK1/2 (1 : 2000), and anti-phospho-ERK1/2 (1 : 2000), at 4°C overnight. Then the membranes were washed and incubated with different HRP-labeled secondary antibodies at room temperature for 1 h. Finally, the proteins were detected using the enhanced chemiluminescence detection reagents (Beyotime Institute of Biotechnology, Jiangsu, China) with a FluorChem M Fluorescent Imaging System (ProteinSimple, Santa Clara, CA, USA). Protein expressions were analyzed using ImageJ software.

### 2.7. Real-Time Quantitative PCR

The expressions of proliferative marker genes and CaSR were examined by real-time quantitative PCR as we previously described [27]. Briefly, total RNA was extracted from pBMSCs by using TRIzol reagent (Invitrogen, Carlsbad, CA, USA) according to the manufacturer's protocol and cDNA was synthesized from 1 *μ*g of total RNA by the M-MLV Reverse Transcriptase (Promega, Madison, WI, USA) and random primers oligo(dT)18 according to the manufacturer's instructions. *β*-actin was used as a candidate housekeeping gene. Real-time quantitative PCR was carried out in Mx3005p instrument (Stratagene, La Jolla, CA, USA) by using SYBR Green Real-Time PCR Master Mix reagents (Toyobo Co., Ltd., Osaka, Japan) and both sense and antisense primers (200 nM for each gene). Relative gene expression of each gene between experimental groups was analyzed using the 2^−ΔΔct^ method. Primer sequences (with their respective PCR fragment lengths) were shown in Table 1.

### 2.8. Statistical Analysis

All data are presented as means ± standard error of the mean (SEM). Statistical analysis was performed using SigmaPlot 12.5 (Systat Software, Inc., San Jose, CA). Differences between means were determined using Student's *t*-test and a confidence level of *P* < 0.05 was considered to be statistically significant.

## 3. Results

### 3.1. [Ca^2+^]_o_ Promoted the Proliferation of pBMSCs and the Expression of CaSR

**Figure 1:**
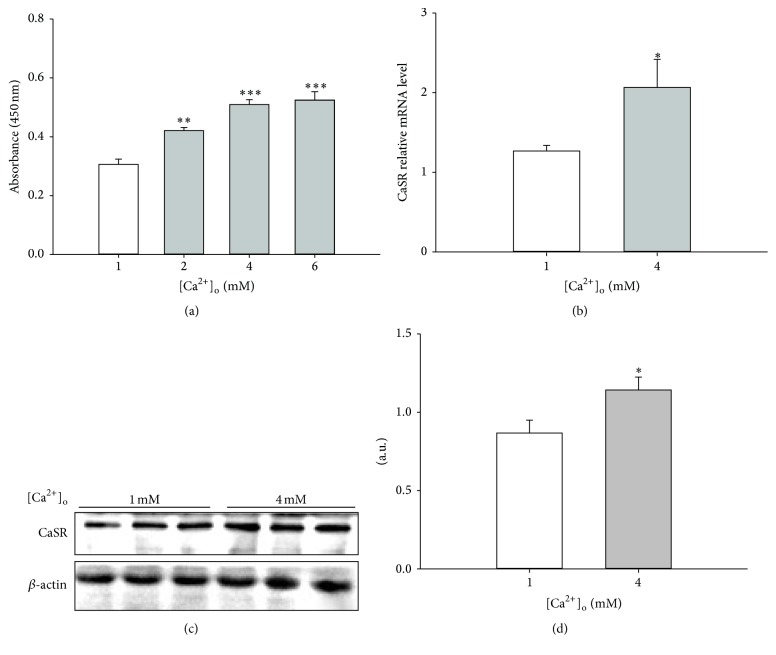
Extracellular calcium ([Ca^2+^]_o_) promoted pBMSCs proliferation and CaSR expression. (a) Effect of various [Ca^2+^]_o_ (1, 2, 4, and 6 mM) on the proliferation of pBMSCs (*n* = 8). (b) Effect of 4 mM [Ca^2+^]_o_ on the mRNA expression of CaSR in pBMSCs after 5-day culture. (c) Western blot analysis of CaSR in pBMSCs after 5-day culture. *β*-actin was used as loading control. (d) Mean ± SEM of immunoblotting bands of CaSR; the intensities of the bands were expressed as the arbitrary units (*n* = 3). ^*∗*^ *P* < 0.05, ^*∗∗*^ *P* < 0.01, and ^*∗∗∗*^ *P* < 0.001 versus 1 mM [Ca^2+^]_o_ group (control).

To assess the effect of [Ca^2+^]_o_ on pBMSCs proliferation, the cells were incubated in DMEM/F12 supplemented with various concentrations (0, 1, 3, and 5 mM) of calcium acetate for 5 days, with the final concentrations of [Ca^2+^]_o_ in the medium up to 1 (control), 2, 4, and 6 mM. The results of CCK-8 assay showed that [Ca^2+^]_o_ significantly stimulated pBMSCs proliferation in a dose-dependent manner, with similar promotive effects observed at 4 and 6 mM [Ca^2+^]_o_ (Figure 1(a)). Thus, 4 mM [Ca^2+^]_o_ was selected and used in our subsequent studies. To determine whether CaSR was involved in [Ca^2+^]_o_-induced pBMSCs proliferation, we examined the effects of [Ca^2+^]_o_ on the expression of CaSR. The result of real-time quantitative PCR indicated that the mRNA expression of CaSR was significantly (*P* < 0.05) enhanced by 4 mM [Ca^2+^]_o_ (Figure 1(b)). Consistently, Western blot results revealed that 4 mM [Ca^2+^]_o_ markedly (*P* < 0.05) increased the protein expression of CaSR (Figures 1(c) and 1(d)). The similar pattern of enhancement of pBMSCs proliferation and CaSR expression implied the possible involvement of CaSR in [Ca^2+^]_o_-induced pBMSCs proliferation.

### 3.2. Inhibition of CaSR Reversed the Promotive Effects of [Ca^2+^]_o_ on pBMSCs Proliferation

**Figure 2:**
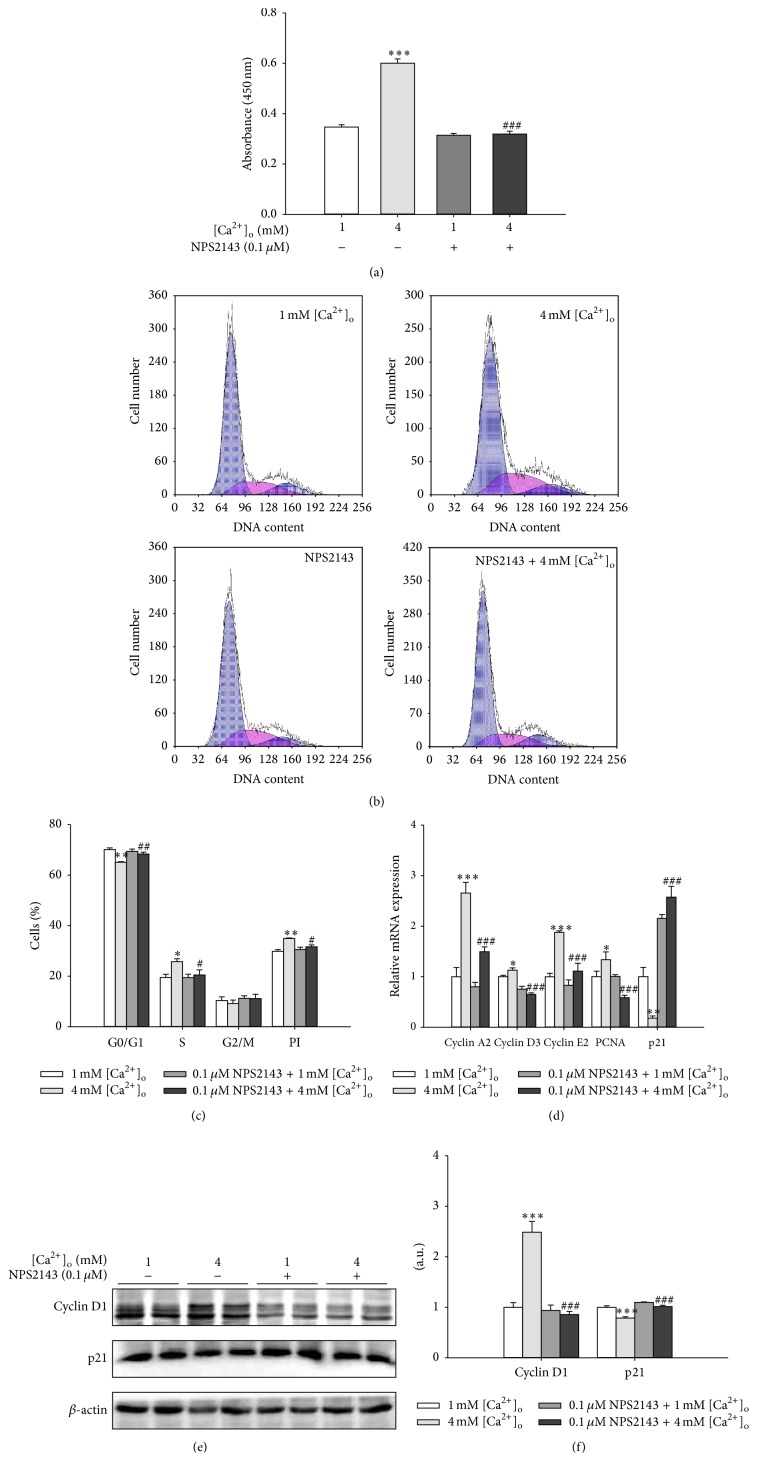
Inhibition of CaSR reversed the promotive effects of [Ca^2+^]_o_ on the proliferation of pBMSCs. (a) Effects of NPS2143 (0.1 *μ*M), an antagonist of CaSR, on the proliferation of pBMSCs after 5-day incubation (*n* = 8). (b) Effects of 4 mM [Ca^2+^]_o_ and/or 0.1 *μ*M NPS2143 on the cell cycle progression of pBMSCs. After pBMSCs were exposed to 4 mM [Ca^2+^]_o_ with or without NPS2143 (0.1 *μ*M) for 5 days, the cells were collected and treated according to the protocol in Materials and Methods. The DNA contents were measured with FACScan flow cytometry. (c) Analysis of proliferation index (PI) and the percentage of cells in G0/G1, S, and G2/M phases. (d) The mRNA expression levels of cyclins (cyclin A2, cyclin D3, and cyclin E2), PCNA, and p21 in response to 4 mM [Ca^2+^]_o_ and/or 0.1 *μ*M NPS2143. (e) Western blot analysis of cyclin D1 and p21 in pBMSCs after 5-day culture. *β*-actin was used as loading control. (f) Mean ± SEM of immunoblotting bands of cyclin D1 and p21; the intensities of the bands were expressed as the arbitrary units (*n* = 4). ^*∗*^ *P* < 0.05, ^*∗∗*^ *P* < 0.01, and ^*∗∗∗*^ *P* < 0.001 versus 1 mM [Ca^2+^]_o_ group (control); ^#^ *P* < 0.05, ^##^ *P* < 0.01, and ^###^ *P* < 0.001 versus 4 mM [Ca^2+^]_o_ group.

To further elucidate the role of CaSR in [Ca^2+^]_o_-stimulated pBMSCs proliferation, NPS2143, a CaSR antagonist, was used to inhibit CaSR in the present study. As shown in Figure 2(a), NPS2143 (0.1 *μ*M) alone had no effect on pBMSCs proliferation. However, NPS2143 significantly (*P* < 0.001) abolished the promotion of pBMSCs proliferation induced by 4 mM [Ca^2+^]_o_. In addition, the results of cell cycle progression analysis via flow cytometry revealed that 4 mM [Ca^2+^]_o_ markedly (*P* < 0.05) increased the proportion of pBMSCs in the S phase and the proliferation index (PI) of pBMSCs and decreased the proportion of pBMSCs in the G0/G1 phase. However, NPS2143 eliminated the effects of [Ca^2+^]_o_ on cell cycle distribution and PI (Figures 2(b) and 2(c)). Furthermore, 4 mM [Ca^2+^]_o_ significantly increased the mRNA expression levels of cyclins (cyclin A2, cyclin D3, and cyclin E2) and PCNA but decreased the mRNA expression of p21, the inhibitor of cyclin-dependent kinase (Figure 2(d)). Moreover, the elevated mRNA expression levels of cyclins and PCNA and the decreased mRNA levels of p21 induced by 4 mM [Ca^2+^]_o_ were reversed by NPS2143. Similarly, the increased protein expression of cyclin D1 and the decreased protein level of p21 induced by 4 mM [Ca^2+^]_o_ were also abolished by NPS2143 (Figures 2(e) and 2(f)). These results showed that the inhibition of CaSR reversed the promotive effects of [Ca^2+^]_o_ on pBMSCs proliferation, thereby indicating the essential role of CaSR in this process.

### 3.3. Elevation of [Ca^2+^]_i_ Level in Response to [Ca^2+^]_o_ Was Blocked by CaSR Inhibition during pBMSCs Proliferation

**Figure 3:**
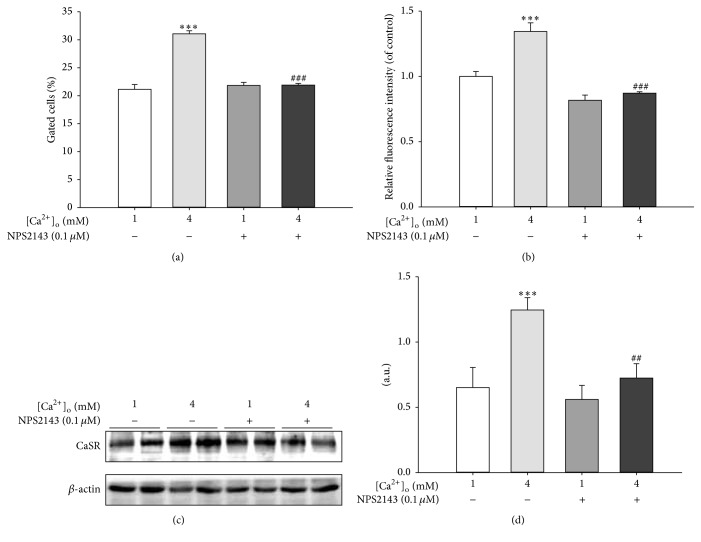
CaSR inhibition blocked the increase of [Ca^2+^]_i_ in response to [Ca^2+^]_o_ during the proliferation of pBMSCs. (a) [Ca^2+^]_i_ of live cells were analyzed by flow cytometry. The percentage of gated positive cells represented the cells stained with Fluo 3-AM. Representation of three independent experiments with similar results. (b) The relative FITC fluorescence intensity of gated positive cells. (c) Western blot analysis of CaSR in pBMSCs after 5-day culture in the presence of 4 mM [Ca^2+^]_o_ and/or 0.1 *μ*M NPS2143. *β*-actin was used as loading control. (d) Mean ± SEM of immunoblotting bands of CaSR; the intensities of the bands were expressed as the arbitrary units (*n* = 4). ^*∗∗∗*^ *P* < 0.001 versus 1 mM [Ca^2+^]_o_ group (control); ^##^ *P* < 0.01 and ^###^ *P* < 0.001 versus 4 mM [Ca^2+^]_o_ group.

The high level of [Ca^2+^]_o_ always resulted in the increase of [Ca^2+^]_i_ levels. In order to confirm the occurrence of this phenomenon and to explore the role of CaSR in this process, we examined [Ca^2+^]_i_ in pBMSCs in the presence of 4 mM [Ca^2+^]_o_ and/or NPS2143 (0.1 *μ*M). As expected, [Ca^2+^]_i_, which was indicated as the percentage of gated cells (Figure 3(a)) or relative fluorescence (Figure 3(b)), was significantly (*P* < 0.001) increased in response to 4 mM [Ca^2+^]_o_, with an increase pattern similar to that of CaSR protein expression induced by 4 mM [Ca^2+^]_o_ (Figures 3(c) and 3(d)). However, as shown in Figures 3(a) and 3(b), the significant elevation of [Ca^2+^]_i_ in response to 4 mM [Ca^2+^]_o_ was blocked by CaSR inhibition with NPS2143, which also abolished the promotive effect of 4 mM [Ca^2+^]_o_ on CaSR protein expression (Figures 3(c) and 3(d)). These data demonstrated that CaSR inhibition blocked the elevation of [Ca^2+^]_i_ induced by 4 mM [Ca^2+^]_o_ in pBMSCs, thereby suggesting that the CaSR-mediated increase in [Ca^2+^]_i_ in response to [Ca^2+^]_o_ might be involved in the [Ca^2+^]_o_-promoted pBMSCs proliferation.

### 3.4. [Ca^2+^]_o_ Activated ERK1/2 Signaling Pathway during the Proliferation of pBMSCs

**Figure 4:**
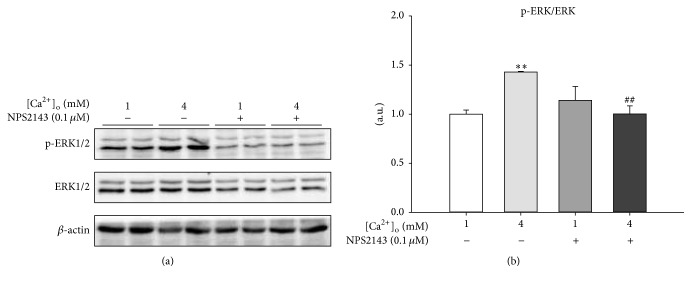
[Ca^2+^]_o_ activated ERK1/2 during the proliferation of pBMSCs. (a) Western blot analysis of phosphor-ERK (p-ERK) and ERK in pBMSCs after 5-day culture. *β*-actin was used as loading control. (b) Mean ± SEM of immunoblotting bands of p-ERK/ERK; the intensities of the bands were expressed as the arbitrary units (*n* = 4). ^*∗∗*^ *P* < 0.01 versus 1 mM [Ca^2+^]_o_ group (control); ^##^ *P* < 0.01 versus 4 mM [Ca^2+^]_o_ group.

Increased [Ca^2+^]_i_ triggered by the high level of [Ca^2+^]_o_ would subsequently activate the downstream intracellular signaling pathway. To explore whether there was a link between CaSR signaling activation and intracellular ERK1/2 signaling pathway, the phosphorylation of ERK1/2 in response to 4 mM [Ca^2+^]_o_ and/or CaSR antagonist NPS2143 were assessed by Western blotting. The results revealed that 4 mM [Ca^2+^]_o_ led to a significant (*P* < 0.01) increase of the p-ERK/ERK ratio, indicating the activation of the ERK1/2 signaling pathway (Figure 4). Meanwhile, the activation of the ERK1/2 signaling pathway induced by [Ca^2+^]_o_ was reversed by NPS2143 (Figure 4). These results indicated that the activation of CaSR signaling and the linked intracellular ERK1/2 signaling pathway might be involved in [Ca^2+^]_o_-stimulated pBMSCs proliferation.

### 3.5. Inhibition of the ERK1/2 Signaling Pathway Abolished [Ca^2+^]_o_-Stimulated pBMSCs Proliferation

**Figure 5:**
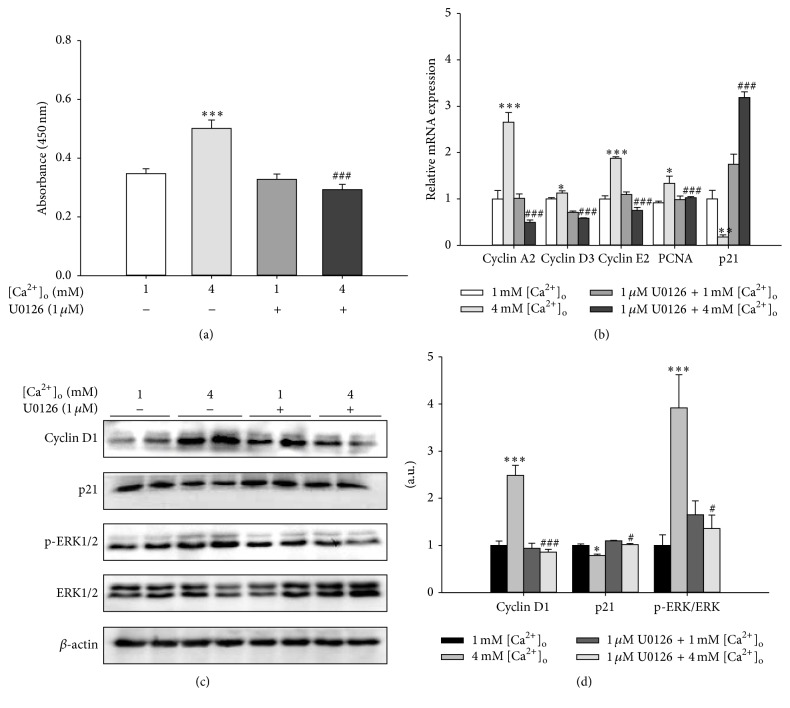
Inhibition of ERK pathway abolished [Ca^2+^]_o_-induced proliferation of pBMSCs. (a) Effects of U0126 (1 *μ*M), an inhibitor of MEK, on the proliferation of pBMSCs after 5-day incubation (*n* = 8). (b) The mRNA expression levels of cyclins (cyclin A2, cyclin D3, and cyclin E2), PCNA, and p21 in response to 4 mM [Ca^2+^]_o_ and/or 1 *μ*M U0126. (c) Western blot analysis of cyclin D1, p21, phosphor-ERK (p-ERK), and ERK in pBMSCs after 5-day culture. *β*-actin was used as loading control. (d) Mean ± SEM of immunoblotting bands of cyclin D1, p21, and p-ERK/ERK. The intensities of the bands were expressed as the arbitrary units (*n* = 4). ^*∗*^ *P* < 0.05 and ^*∗∗∗*^ *P* < 0.001 versus 1 mM [Ca^2+^]_o_ group (control); ^#^ *P* < 0.05 and ^###^ *P* < 0.001 versus 4 mM [Ca^2+^]_o_ group.

To further verify the role of ERK1/2 signaling pathway in [Ca^2+^]_o_-induced cell proliferation of pBMSCs, U0126, a selective inhibitor of ERK kinase (MEK1/2), was applied to inhibit the kinase activity of MEK1/2 and thus to prevent the activation of ERK1/2 in our study. We determined that the significant increase of p-ERK/ERK ratio in response to 4 mM [Ca^2+^]_o_ was reversed by U0126 (Figures 5(c) and 5(d)). The findings of CCK-8 assay showed that U0126 (1 *μ*M) alone had no effect on the proliferation of pBMSCs. However, U0126 could eliminate the promotion of pBMSCs proliferation induced by 4 mM [Ca^2+^]_o_ (Figure 5(a)). In agreement, the significant increase in the mRNA levels of cyclin A2, cyclin D3, cyclin E2, and PNCA and the significant decrease of p21 mRNA levels induced by 4 mM [Ca^2+^]_o_ were also abolished by U0126 (Figure 5(b)). In addition, U0126 reversed the effects of [Ca^2+^]_o_ on the protein expression of cyclin D1 and p21 (Figures 5(c) and 5(d)). These results strongly suggested that [Ca^2+^]_o_ promoted pBMSCs proliferation through the activation of the ERK1/2 signaling pathway.

## 4. Discussion

In the present study, we determined that [Ca^2+^]_o_ promoted pBMSCs proliferation by regulating cell cycle progression and the expression levels of proliferative marker genes through the activation of plasma membrane receptor CaSR and intracellular ERK1/2 signaling pathway. The pro-proliferation effects of [Ca^2+^]_o_ have been reported in various cells such as rat bone marrow-derived progenitor cells [9], osteoblasts [10], preadipocytes [11], and porcine synovium-derived mesenchymal stromal cells [12]. In line with these results, we found that [Ca^2+^]_o_ promoted pBMSCs proliferation in a dose-dependent manner, with the similar promotive effects observed when [Ca^2+^]_o_ was greater than or equal to 4 mM. However, Liu et al. reported that the optimal [Ca^2+^]_o_ needed for rabbit BMSCs to proliferate was 1.8 mM and the higher level of [Ca^2+^]_o_ did not change cell proliferation [28]. In addition, it was demonstrated that the high level of [Ca^2+^]_o_ (7 or 10 mM) slowed the rate of porcine osteoblasts proliferation [29]. Furthermore, Lin et al. found that low calcium (0.09 mM) greatly enhanced the growth rate and extended the lifespan of human adipose-derived MSCs [30]. These discrepant effects of [Ca^2+^]_o_ on BMSCs proliferation might be attributed to the various species and/or culture conditions. In addition, the heterogeneous characteristic of BMSCs should also be considered for the different effects of [Ca^2+^]_o_ on BMSCs proliferation. The pBMSCs used in our study were positive for mesenchymal surface markers CD29 and CD44 and negative for hematopoietic marker CD45 and for the adhesion molecule CD31 and were able to differentiate into adipocytes and myocytes [4], which represented only a subpopulation of MSCs in porcine bone marrow. It should be noted that this study was performed in 20% oxygen tensions, which are generally used in standard culture. It has been reported that, in the presence of high oxygen tension (20% to 21% O_2_) culture conditions, MSCs derived from human [31], mouse [32], and rat [33] show lower proliferative activity than that in the presence of low oxygen tension (2% to 5% O_2_) culture conditions. Thus, the effects of [Ca^2+^]_o_ on pBMSCs proliferation in low oxygen tension should be further investigated in future study.

The cell cycle progression and the expression levels of proliferative marker genes (cyclins, PCNA, and p21) were detected to elucidate the stimulatory effects of [Ca^2+^]_o_ on pBMSCs proliferation. In our study, we determined that [Ca^2+^]_o_ significantly decreased the ratio of G0/G1 phase and increased the percentage of the S phase and the PI in pBMSCs, compared with those of control group. These data indicated that [Ca^2+^]_o_ accelerated cell cycle progression from the G0/G1 phase to the S phase and promoted pBMSCs proliferation. It has been implicated that cyclin D and cyclin E are required for the transition from G1 to S phase of the cell cycle, whereas cyclin A is involved in the initiation and completion of DNA replication during the S phase [34, 35]. In accord, we detected the elevated expressions of cyclin A2, cyclin D1/3, and cyclin E2 induced by [Ca^2+^]_o_. In addition, the level of PCNA, an essential component for DNA replication machinery [36], was also improved by [Ca^2+^]_o_. By contrast, p21, the inhibitor of cyclin-dependent kinase, was inhibited in the presence of [Ca^2+^]_o_. Thus, these findings indicated that [Ca^2+^]_o_ stimulated pBMSCs proliferation by influencing the cell cycle progression and the expression levels of proliferative marker genes.

[Ca^2+^]_o_ elicits its effects on the regulation of cell proliferation either via calcium influx through calcium channels or by activating CaSR-mediated signaling pathway [13]. Although it has been reported that the voltage-gated calcium channels (VGCCs) antagonist nifedipine exerts antiproliferative effects on rats BMSCs [37], our result revealed that nifedipine had no effect on [Ca^2+^]_o_-stimulated pBMSCs proliferation (see the Supplementary Material available online at http://dx.doi.org/10.1155/2016/6570671). In contrast, we found the expression of CaSR in pBMSCs, which was also expressed in mesangial cells [15], osteoblasts [10], preadipocytes [11], and rat BMSCs [38] and played a vital role in the regulation of cell proliferation. In addition, the inhibition of CaSR by NPS2143 reversed the promotive effects of [Ca^2+^]_o_ on pBMSCs proliferation, suggesting the involvement of CaSR in [Ca^2+^]_o_-promoted pBMSCs proliferation. In agreement, it has been demonstrated that activation of CaSR by [Ca^2+^]_o_ contributes to the cell proliferation of human mesangial cells [15] and osteoblasts [10]. In addition, CaSR activation with its agonist increased the cell proliferation of human aortic smooth muscle cells, while CaSR knockdown reduced the cell proliferation [39]. However, Rey et al. described that [Ca^2+^]_o_-induced stimulation of CaSR inhibited the cell proliferation of human colon epithelial cells [40]. The opposite effects of CaSR activation on cell proliferation might be caused by the distinguished cell types and varied physiological environment.

A large body of evidence has determined that [Ca^2+^]_o_ can induce [Ca^2+^]_i_ increment [10, 15, 41]. Consistently, our results showed a significant elevation of [Ca^2+^]_i_ in response to [Ca^2+^]_o_, which was reversed by CaSR inhibition with NPS2143. These finding indicated the involvement of CaSR in the [Ca^2+^]_o_-induced increase in [Ca^2+^]_i_ during pBMSCs proliferation. Notably, it was reported that [Ca^2+^]_i_ can promote cyclin E expression and the initiation of DNA synthesis at G1/S transition, thereby contributing to the completion of the cell cycle [42]. Our results also showed that CaSR inhibition by NPS2143 reversed the promotion of pBMSCs proliferation, the change of cell cycle distribution, and the expression levels of proliferative marker genes (cyclins, PCNA, and p21), which were induced by the high level of [Ca^2+^]_o_. Together, these observations give rise to the idea that the [Ca^2+^]_o_-induced elevation of [Ca^2+^]_i_ and the enhancement of pBMSCs proliferation were mediated, at least in part, by activation of CaSR.

Numerous studies have shown that the activation of the ERK1/2 signaling pathway is involved in regulating the proliferation of various cells, including kidney epithelial cells [18], smooth muscle cell [19], melanoma cell [20], and preadipocytes [11]. Accordingly, in the present study, the ERK1/2 signaling pathway was activated by [Ca^2+^]_o_ and the activation of ERK1/2 was abolished by NPS2143. In addition, the inhibition of ERK1/2 signaling pathway with U0126 reversed the stimulation of pBMSCs proliferation, the alteration of cell cycle distributions, and the expression levels of cell cycle marker genes, which were induced by [Ca^2+^]_o_. These results provided the evidence that [Ca^2+^]_o_ stimulated pBMSCs proliferation, at least in part, via activation of CaSR and the linked intracellular ERK1/2 signaling pathway. However, Tfelt-Hansen et al. found that the activation of p38 MAPK and PI3K but not that of ERK1/2 by CaSR promoted the cell proliferation of rat Leydig cancer cells [43]. The reason for the discrepancy between the study of Tfelt-Hansen et al. and our study might be the different cell types.

In conclusion, our findings demonstrated that CaSR was expressed in pBMSCs and that the enhanced proliferation of pBMSCs in response to [Ca^2+^]_o_ was associated with activation of plasma membrane receptor CaSR, elevation of [Ca^2+^]_i_, and the enhancement of the intracellular ERK1/2 signaling pathway. These data may be useful for the application of pBMSCs in future clinical studies aimed at tissue regeneration and repair.

## Supplementary Material

Supplementary Figure: Nifedipine (1 μM), an antagonist of VGCC, has no effect on the promotion of pBMSCs proliferation induced by 4 mM [Ca^2+^]_o_.

## Figures and Tables

**Table 1:** The primer sequences used for real-time quantitative PCR.

Gene	Forward (5′-3′)	Reverse (5′-3′)	Amplification length (bp)
*β*-actin	TGTCATGGACTCTGGGGATG	GTGGTGGTGAAGCTGTAGCC	156
CaSR	GTGCCATAGAGGAAATAAACAG	CCACGGCGATGGTAGAG	198
Cyclin A2	CCTTGGAAAGCAAACAGTAAAC	TTGGTCCAGGTAAAGTAACAGC	148
Cyclin D3	GGTCCTGGGGAAGCTCAAGT	CATGGCAAAGGTGTAATCTGTA	163
Cyclin E2	AAACACCCCACAAAGAAATAGG	CCCAGCTTAAATCAGGCAAA	105
PNCA	GATGCTGTTGTAATTTCCTGTG	CTCTATGGTAACTGCTTCCTCC	129
p21	AGGACCATGTGGACCTGTTG	TTAGGGCTTCCTCTTGGAGA	173
