# Supplementary material for: Enhanced Proliferation of Porcine Bone Marrow Mesenchymal Stem Cells Induced by Extracellular Calcium is Associated with the Activation of the Calcium-Sensing Receptor and ERK Signaling Pathway
Source: Stem Cells Int. 2016 Mar 30;2016:6570671. doi: 10.1155/2016/6570671 (PMC4829725; doi:10.1155/2016/6570671)

Supplementary material

1, Effects of Nifedipine (1 μM), an antagonist of VGCC, on the proliferation of pBMSCs after 5-day incubation.

The result shows that nifedipine has no effect on the promotion of pBMSCs proliferation induced by 4 mM [Ca^2+^]_o_. ****P* < 0.001 versus 1 mM [Ca^2+^]_o_ group (control).


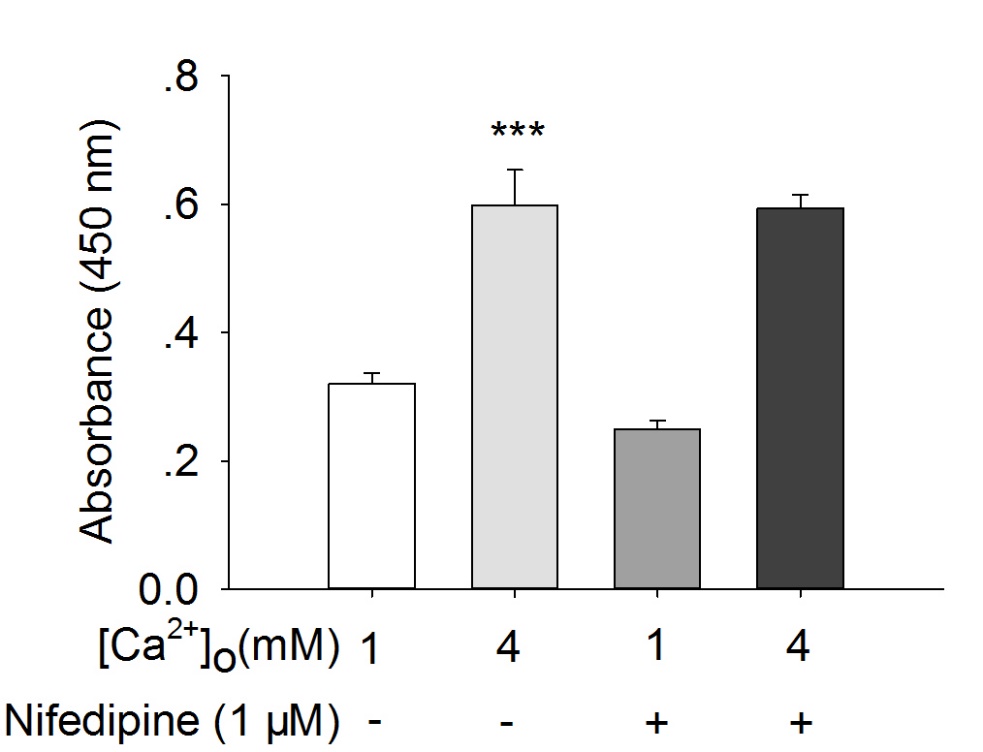

Supplement: Supplementary file 1 — Supplementary Figure: Nifedipine (1 μM), an antagonist of VGCC, has no effect on the promotion of pBMSCs proliferation induced by 4 mM [Ca2+]o. [file 6570671.f1.docx]
